# Integrin αVβ3 antagonist-c(RGDyk) peptide attenuates the progression of ossification of the posterior longitudinal ligament by inhibiting osteogenesis and angiogenesis

**DOI:** 10.1186/s10020-024-00822-x

**Published:** 2024-05-02

**Authors:** Xiangwu Geng, Yifan Tang, Changjiang Gu, Junkai Zeng, Yin Zhao, Quanwei Zhou, Lianshun Jia, Shengyuan Zhou, Xiongsheng Chen

**Affiliations:** Spine Center, Department of Orthopaedics, Changzheng Hospital, Naval Medical University (Second Military Medical University), Shanghai, 200003 China

**Keywords:** OPLL, Osteogenesis, Angiogenesis, Integrin αVβ3, c(RGDyk)

## Abstract

**Background:**

Ossification of the posterior longitudinal ligament (OPLL), an emerging heterotopic ossification disease, causes spinal cord compression, resulting in motor and sensory dysfunction. The etiology of OPLL remains unclear but may involve integrin αVβ3 regulating the process of osteogenesis and angiogenesis. In this study, we focused on the role of integrin αVβ3 in OPLL and explored the underlying mechanism by which the c(RGDyk) peptide acts as a potent and selective integrin αVβ3 inhibitor to inhibit osteogenesis and angiogenesis in OPLL.

**Methods:**

OPLL or control ligament samples were collected in surgery. For OPLL samples, RNA-sequencing results revealed activation of the integrin family, particularly integrin αVβ3. Integrin αVβ3 expression was detected by qPCR, Western blotting, and immunohistochemical analysis. Fluorescence microscopy was used to observe the targeted inhibition of integrin αVβ3 by the c(RGDyk) peptide on ligaments fibroblasts (LFs) derived from patients with OPLL and endothelial cells (ECs). The effect of c(RGDyk) peptide on the ossification of pathogenic LFs was detected using qPCR, Western blotting. Alkaline phosphatase staining or alizarin red staining were used to test the osteogenic capability. The effect of the c(RGDyk) peptide on angiogenesis was determined by EC migration and tube formation assays. The effects of the c(RGDyk) peptide on heterotopic bone formation were evaluated by micro-CT, histological, immunohistochemical, and immunofluorescence analysis in vivo.

**Results:**

The results indicated that after being treated with c(RGDyk), the osteogenic differentiation of LFs was significantly decreased. Moreover, the c(RGDyk) peptide inhibited the migration of ECs and thus prevented the nutritional support required for osteogenesis. Furthermore, the c(RGDyk) peptide inhibited ectopic bone formation in mice. Mechanistic analysis revealed that c(RGDyk) peptide could inhibit osteogenesis and angiogenesis in OPLL by targeting integrin αVβ3 and regulating the FAK/ERK pathway.

**Conclusions:**

Therefore, the integrin αVβ3 appears to be an emerging therapeutic target for OPLL, and the c(RGDyk) peptide has dual inhibitory effects that may be valuable for the new therapeutic strategy of OPLL.

**Supplementary Information:**

The online version contains supplementary material available at 10.1186/s10020-024-00822-x.

## Introduction

Ossification of the posterior longitudinal ligament (OPLL) caused by ectopic bone can cause compression on the spinal cord or nerve roots, resulting in severe neurological dysfunction (Saetia et al. 2011). The incidence of OPLL is about 1.9-4.3% in some Eastern Asian populations, but it is rarely seen in the Caucasians (Matsunaga and Sakou 2012). Severe OPLL can lead to paralysis in patients, causing a huge socioeconomic burden to patients and society. The pathogenesis of OPLL has not been fully elucidated, but genetic and environmental factors have been implicated in the aetiology of OPLL (Ikegawa 2014; Nakajima et al. 2014; Nam et al. 2019). Although conservative treatments have focused on suppressing inflammation and controlling pain, treatment targeting the ossification of ligaments is insufficient. In addition, applying a variety of surgical strategies for OPLL exerts limited therapeutic effects with frequent complications to data (Le et al. 2022). Thus, more precise therapeutic targets with less negative effects are imperative.

The posterior longitudinal ligament, located at the posterior edge of the vertebral body, is involved in maintaining stability and balance of the spine and is subject to long-term stress. In our previous in vivo experimental study, cyclic tensile stress promoted the ossification of the ligament flavum in rats and confirmed that the expression of integrin β3 and VEGF was upregulated (Zhao et al. 2021; Gao et al. 2020). In short, it has been shown literature that exterior stress stimulation can affect osteoblast differentiation of stem cells. Interestingly, the interaction between integrin and extracellular matrices is essential for mechanotransduction (Ross et al. 2013; Schwartz 2010; Case and Waterman 2015). Extensive evidence indicates that mechanical stimulation regulates integrin activation (Kechagia et al. 2019; Cooper and Giancotti 2019; Sun et al. 2019). The literature reports that the activation of integrin induces osteogenic differentiation of human fibroblasts (Peng et al. 2021). Ossification of ligament fibroblasts (LFs) was shown to contribute to OPLL (Liu et al. 2020; Xu et al. 2022). LFs, as an undifferentiated mesenchymal cell subset, are thought to exist within this population and play a crucial role in osteoblast differentiation (Chen et al. 2018; Su et al. 2018; Cho et al. 2017). Furthermore, endothelial cells (ECs) can secrete various cytokines to promote osteoblast formation and are essential for maintaining LF niches (Salhotra et al. 2020).

Integrins are heterodimeric adhesion receptors that mediate extracellular matrix interaction and are believed to control intracellular signalling pathways as mechanoreceptors. Integrin is composed of an α-subunit and a β-subunit bound in a noncovalent complex with the ligand-binding site on the membrane surface. In recent years, the most studied integrin has focused on integrin αVβ3 for cancer, orthopaedics, and cardiovascular disease (Lecker et al. 2021; Fu et al. 2020; Liu et al. 2018; Tang et al. 2021b). However, whether integrin αVβ3 plays an important role in ectopic bone formation in OPLL is still unknown.

In our study, we found that integrin αVβ3 was significantly upregulated in OPLL samples and primary LFs obtained from OPLL. The binding of integrins with ligands is achieved through recognition of the RGD sequence on the ligand, thus the search for suitable peptides carrying the RGD sequence that selectively bind to integrin αVβ3 may enable targeted inhibition of integrin αVβ3. Here, we reported the cyclo (Arg-Gly-Asp-D-Tyr-Lys) peptide, which exhibits high affinity for integrin αVβ3. We explored the mechanism by which c(RGDyk) inhibited osteogenesis and angiogenesis in OPLL to provide more insight into the mystery of OPLL.

## Materials and methods

### Sample collection and cell culture

With the approval of the Ethics Committee of our institution (Naval Medical University) and patient consent, the ligament samples were collected from patients diagnosed with OPLL(who underwent anterior cervical corpectomy and fusion)or non-OPLL (cervical spine trauma patients who underwent anterior cervical corpectomy and fusion) during surgeries. Sixteen patients (8 with OPLL and 8 with non-OPLL) were included between August 2019 and July 2020. Our professional spine surgeons confirmed the diagnosis of OPLL or cervical trauma by preoperatively presenting the patient’s symptoms, signs, and imaging data. Moreover, detailed information on the participants is listed in additional file 1: Table S1. Ligament cell culture from patients with primary OPLL or non-OPLL has been previously described (Tang et al. 2022). Briefly, approximately 1 mm^3^ pieces of sample tissue were plated on Petri dishes with high-glucose DMEM (H-DMEM, HyClone, USA) with 10% FBS (Gibco, USA) and 1% penicillin/streptomycin (Gibco, USA); the tissue pieces were then incubated at 37 ℃ under 5% CO_2_. After approximately 10 days, a mass of cells with spindle or flattened shapes emerged around the tissue pieces and grew adherently. When the bottom of the dish was 70-80% covered with cells, the cells were harvested for serial passage and analysis. LFs from passages 1–3 were chosen for subsequent experiments.

The ECs used in this experiment were obtained from the Cell Bank of the CAS (Chinese Academy of Sciences, Shanghai, China). The ECs were cultured in endothelial cell Medium (ECM, Cyagen Biosciences, Guangzhou, China) containing endothelial cell growth supplement and 5% FBS in an incubator at 37 ℃ under 5% CO_2_. ECs from passages 1–3 were chosen for subsequent experiments.

### Flow cytometry

To identify the mesenchymal characteristics of LFs, we used flow cytometry for surface characterization. Isolated primary LFs with a confluence of 80% were chosen for subsequent experiments. Simply, after the cells were digested with 0.25% trypsin, the cells were collected by centrifugation, adjusted to a density of 1 × 10^6^/ml, and finally added to the suspension in 100 µL of PBS buffer with 2% FBS for washing three times. Subsequently, FITC-conjugated antibodies were added, including CD34 (34-581-01, eBioscience, USA), CD45 (MHCD4504, eBioscience, USA), CD90 (17-0909-42, eBioscience, USA), and CD105 (MA1-19594, eBioscience, USA), followed by incubation in the dark for 30 min. After that, the incubated cells were centrifuged and washed with PBS. Finally, the cells were detected with a flow cytometer (Cyan ADP, Beckman Coulter, USA), and the positive rate of surface antigen was analysed and calculated by FlowJo software.

### Osteogenic, adipogenic, and chondrogenic differentiation

For osteogenic differentiation, ligament fibroblasts derived from patients with OPLL (500 µl, 2 × 10^4^ cells/ml) were seeded into 24-well plates (Corning, USA) and incubated at 37 ℃ under 5% CO_2_. When the LF confluence reached 80–90%, the medium was replaced with osteogenic induction medium (HUXMA-90,021, Cyagen, China). The medium of the cells was refreshed every two days. After osteogenic induction, the cells were stained by using an ALP staining kit (P0321S, Beyotime, China) on Day 7 and 2% Alizarin Red S (SCR028, Sigma-Aldrich, USA) on Day 21.

For adipogenic differentiation, LFs (1 ml, 5 × 10^4^ cells/ml) were seeded in 12-well plates (Corning, USA) and incubated at 37 ℃ under 5% CO_2_. When the LF confluence reached 100%, the medium was replaced with adipogenic differentiation medium (HUXXC-90,031, Cyagen, China). The adipogenic differentiation medium was divided into liquid A and liquid B. Liquid A is mainly used to induce the formation of lipid droplets, while liquid B is mainly used to maintain the morphology of lipid droplets. The cells were cultured with liquid A for three days and then replaced with liquid B for one day. After 21 days, the cells were stained with Oil Red O (OILR-10,001, Cyagen, China) and photographed with a microscope.

For chondrogenic differentiation, LFs reaching 80% confluence were first digested with trypsin and then centrifuged at 220 g for 5 min, discarding the supernatant. Subsequently, the cells were resuspended in an OriCell® chondrogenic differentiation medium (HUXMX-90,041, Cyagen, China), and the cell density was adjusted to 2 × 10^6^ cells/ml. Next, 1 ml of cell suspension was aspirated and seeded into a 15 ml centrifuge tube, followed by another centrifugation step. After centrifugation, the centrifuge tube cap was loosened, and the tube was gently placed vertically in a 37 ℃, 5% CO_2_ incubator. After 24 h of induction, cell aggregates could be observed at the bottom of the centrifuge tube. Every two days, the old medium was carefully aspirated, and 1 ml of fresh medium was added. After 21 days of induction culture, chondrospheres were formed. To further observe the morphology of the chondrospheres, they were fixed with 4% paraformaldehyde, sectioned, and stained with 2% Alcian Blue (TMS-010, Sigma-Aldrich, USA), followed by microscopic examination and recording of the results.

### Cell proliferation assay

First, the medium was prepared with different of c(RGDyk) peptides (NJPeptide, Nanjing, China) for the following experiment. Next, LFs and ECs were cultured in medium with different concentrations of c(RGDyk) for one day. Cell proliferation was tested with a Cell Counting Kit-8 assay (CCK-8, C0038, Beyotime, Shanghai, China). Briefly, after cells were incubated in a 10% CCK-8 solution for 1 h at 37 ℃, OD_450_ values were detected by a spectrophotometric microplate reader.

### Immunofluorescence (IF) assay

LFs or ECs (500 µl, 2 × 10^4^ cells/ml) were seeded in 24-well plates and incubated with different concentrations of c(RGDyk) at 37 ℃ under 5% CO_2_. For cell IF analysis, cells were fixed with 4% formaldehyde and then washed three times with PBS, followed by permeabilization with 0.5% Triton X-100 for 5 min. After blocking with 5% bovine serum albumin (BSA) for 1 h, the cells were incubated overnight at approximately 16 h at 4 ℃ with anti-integrin αVβ3 (1:200, BS-1310R, Bioss, USA). After incubation, the cytoskeleton actin of cells was stained by FITC-phalloidin (1:200, YP0059S, Bioscience, Shanghai) for 20 min. Subsequently, secondary antibodies were used, and the cell nuclei were stained by DAPI (P0131, Beyotime, Shanghai, China) for 5 min. For tissue IF analysis, first, the decalcified OPLL or scaffold samples were fixed in 4% paraformaldehyde, dehydrated in a graded ethanol series, vitrified using dimethylbenzene, and then embedded in paraffin. Subsequently, the paraffin sections were deparaffinized by xylene and hydrated in an alcohol gradient. The tissue IF analysis procedures were similar to those of cell IF. In brief, sections were incubated overnight at 4 ℃ with anti-CD31 (1:1000, 3528 S, CST, USA). After incubation, sections were incubated with secondary antibody (1:100, AS037, ABclonal, China) for 2 h and DAPI for 5 min at room temperature. Finally, fluorescence microscopy was used to photograph the cells.

### Cell migration assay

Transwell and scratch assays were used to evaluate the effects of c(RGDyk) on the migration property of ECs. Cell proliferation experiments have demonstrated that when the concentration of the c(RGDyk) exceeds 10 µM, cell activity is inhibited, exhibiting a certain level of toxicity. Therefore, we selected peptide concentrations of 1, 5, and 10 µM for investigating cell migration assay. We prepared a few 24-well transwell plates with 8-µm pores for the transwell assay. First, we seeded the ECs (800 µL, 2 × 10^4^ cells/ml) in the upper chambers and preincubated them with different concentrations of c(RGDyk) for 1 h. Second, we incubated them with the medium in the lower chambers for 24 h with c(RGDyk) remaining in the upper chambers. Finally, after the end of incubation, the migrated ECs were fixed with 10% paraformaldehyde for 20 min, washed with PBS solution, stained with 0.5% crystal violet (Beyotime, China), and photographed with a microscope.

For the scratch assay, ECs were seeded in 6-well plates (Corning, USA). In brief, the cells were scratched with a sterile 200 µL pipette tip perpendicular to the bottom of the plate after reaching 90% confluence. At 0 h and 24 h after scratching, the cells were photographed and analysed using a light microscope and ImageJ software. The width range of the EC movement was measured by ImageJ software. The calculation method of EC migration area (A) was as follows: A=(A_1−_A_2_)/A_1_ × 100%, where A_1_ is the initial area and A_2_ is the residual area of the wound.

### Tube formation assay

The tube formation assay was performed to evaluate the effect of c(RGDyk) on the angiogenic ability of ECs. Briefly, 60 µL per well of Matrigel (Corning, USA) with different concentrations of c(RGDyk) was spread gently into a 96-well plate (Corning, USA) and then incubated in a 37 ℃ incubator for 50 min. Afterwards, ECs at a density of 2 × 10^4^ cells per well were seeded in each well. After incubation for 6 h, the cells were photographed and analysed using a light microscope and ImageJ software. The formed nodes and meshes were measured.

### RNA extraction, RNA-sequencing (RNA-seq), and qPCR

Firstly, cultured LFs from patients with primary OPLL or non-OPLL were lysed with TRIzol (Invitrogen, USA) to isolate total RNA. Subsequently, a cDNA library was constructed for RNA-seq to analyze the expression of differentially expressed genes. Finally, total RNA was reverse transcribed into cDNA using a ReverTra Ace® qPCR RT Kit (Toyobo, Japan) following the manufacturer^’^s instructions. q-PCR was performed with the SYBR Green Mix, and the relative expression of genes, including integrin αV, α2, α3, α4, α2b, α9, αL, β3, β4, β8, ALP, RUNX2, OCN, OPN, and Collagen I, was quantified with normalization to β-actin mRNA. The sequences of the primers are shown in additional file 1: Table S2.

### Synthesis and cell transfection of siRNA

The siRNAs targeting Integrin αV and Integrin β3 were designed and synthesized by Genomeditech Cor (Shanghai, China). LF transfection during osteoblastogenesis in vitro was performed on the first day after osteogenic differentiation induction. The siRNA and GMTrans Liposomal Transfection Reagent (Genomeditech, Shanghai, China) were mixed according to the manufacturer’s guidelines and added to LF culture, followed by 24 h of incubation. The culture medium was changed every day until the LFs were harvested for detection. The siRNA sequences are shown in additional file 1: Table S3.

### Western blots

First, cells were lysed in ice-cold lysis buffer (KeyGEN BioTECH, China) containing protease and phosphatase inhibitors. After the lysate was centrifuged at 12,000 × g for 10 min at 4 ℃, cell proteins were extracted from the supernatant. Next, a 4x loading buffer was added to the proteins of different groups, and the samples were boiled for 10 min before electrophoresis. While the protein samples were separated by SDS‒PAGE, the proteins were transferred to PVDF membranes. Second, protein samples were incubated with primary antibodies overnight at approximately 16 h at 4 ℃. The following primary antibodies were used: Anti-GAPDH (1:1000, GB15002, Servicebio, China), Anti-β-actin (1:1000, GB15003, Servicebio, China), Anti-ALP (1:1000, ab229126, Abcam, Cambridge, UK), Anti-RUNX2 (1:1000, ab264077, Abcam, Cambridge, UK), Anti-OCN (1:1000, ab93876, Abcam, Cambridge, UK), Anti-OPN (1:1000, ab214050, Abcam, Cambridge, UK), Anti-Collagen I (1:1000, ab138492, Abcam, Cambridge, UK), Anti-integrin αVβ3 (1:500, bs-1310R, Bioss, USA), Anti-FAK (1:1000, A11131, ABclonal, China), Anti-ERK1/2 (1:1000, 4696 S, CST, USA), Anti-p-VEGFR2 (1:1000, 2478 S, CST, USA), Anti-p-FAK (1:500, AP0302, ABclonal, China), and Anti-p-ERK1/2 (1:1000, 8544 S, CST, USA). Third, the membranes were incubated with species-matched secondary antibodies (1:1000, AS081, ABclonal, China) for 1 h at room temperature. Finally, the target proteins were visualized by the enhanced chemiluminescence (ECL, Millipore, USA) detection system. Original immunoblot pictures of western blot are shown in additional file 1: Figures S5, S6, and S7.

### Histology and immunohistochemical (IHC) assays

First, paraffin sections used for histology and immunohistochemical assays were the same as described above for the tissue IF assay. Next, the sections were stained with standard HE staining protocols. For the IHC assay, sections were subsequently incubated overnight at 4 ℃ with primary antibodies. The following primary antibodies were used: anti-OCN (1:150, ab93876, Abcam, USA), and anti-integrin αVβ3 (1:200, bs-1310R, Bioss, USA). Finally, sections were scanned by moving rapidly under the microscope of the image scanner (3DHISTECH, Hungary).

### In vivo heterotopic bone formation assay

In an in vitro assay, we proved that 10 µM is the best concentration of c(RGDyk) to inhibit osteogenesis and angiogenesis. Thus, we used a 10 µM c(RGDyk) for in vivo ectopic bone formation assays. First, under osteogenic induction conditions, LFs were pretreated with a concentration of 10 µM c(RGDyk) cyclic peptide for 5 days. Then, the LFs were cocultured with Bio-Oss Collagen scaffolds (Geistlich, GEWO GmbH, Germany) in osteogenic medium with or without c(RGDyk) (10 µM) for 48 h. Next, the mixture of scaffold and LFs was subcutaneously implanted on the back of nude mice (4-week-old BALB/c male nude, Shanghai Legen Biotechnology Co., Ltd., China) as described previously (Tang, Sun 2022). After 8 weeks, the scaffolds implanted in the animals were retrieved and fixed in 10% formalin solution for 48 h, followed by decalcification in 10% EDTA (BB-23,613, BestBio, China) solution for 4 weeks. The decalcified bone specimens were embedded in paraffin, and tissue blocks of paraffin were then sectioned at a thickness of 5 μm for subsequent experiments. In this study, nude mice were randomly assigned to three distinct groups: the NC group, the LFs group, and the LFs-RGDyk group. The NC group served as the blank control group, in which mice received implantation of scaffolds without any additional treatment. The LFs group was designated as the experimental control group, where mice were implanted with cell-loaded scaffolds. The LFs-RGDyk group served as the experimental group, in which mice were implanted with a mixture of scaffolds pre-treated with RGDyk. Preoperative, intraoperative, and postoperative images of animal models are shown in Additional file 1: Figure S1.

### Micro-CT

To determine whether c(RGDyk) inhibits new bone formation in vivo, we conducted micro-CT scans on the bioscaffolds harvested after treatment at 8 weeks after the surgery. Finally, the 3D reconstruction image was performed using ReconDaemon software (Pingseng Healthcare Ctvox, Shanghai), and data analysis was performed using the Avatar3 software (Pingseng Healthcare Ctvox, Shanghai).

### Statistical analysis

All experimental data are presented as the mean value ± SD. Student’s t test or one-way analysis of variance (ANOVA) was used to assess independent variables appropriately. A *P < 0.05 was considered to be statistically significant.

## Results

### Integrin αVβ3 is upregulated in the ligaments of patients with OPLL

**Fig. 1:**
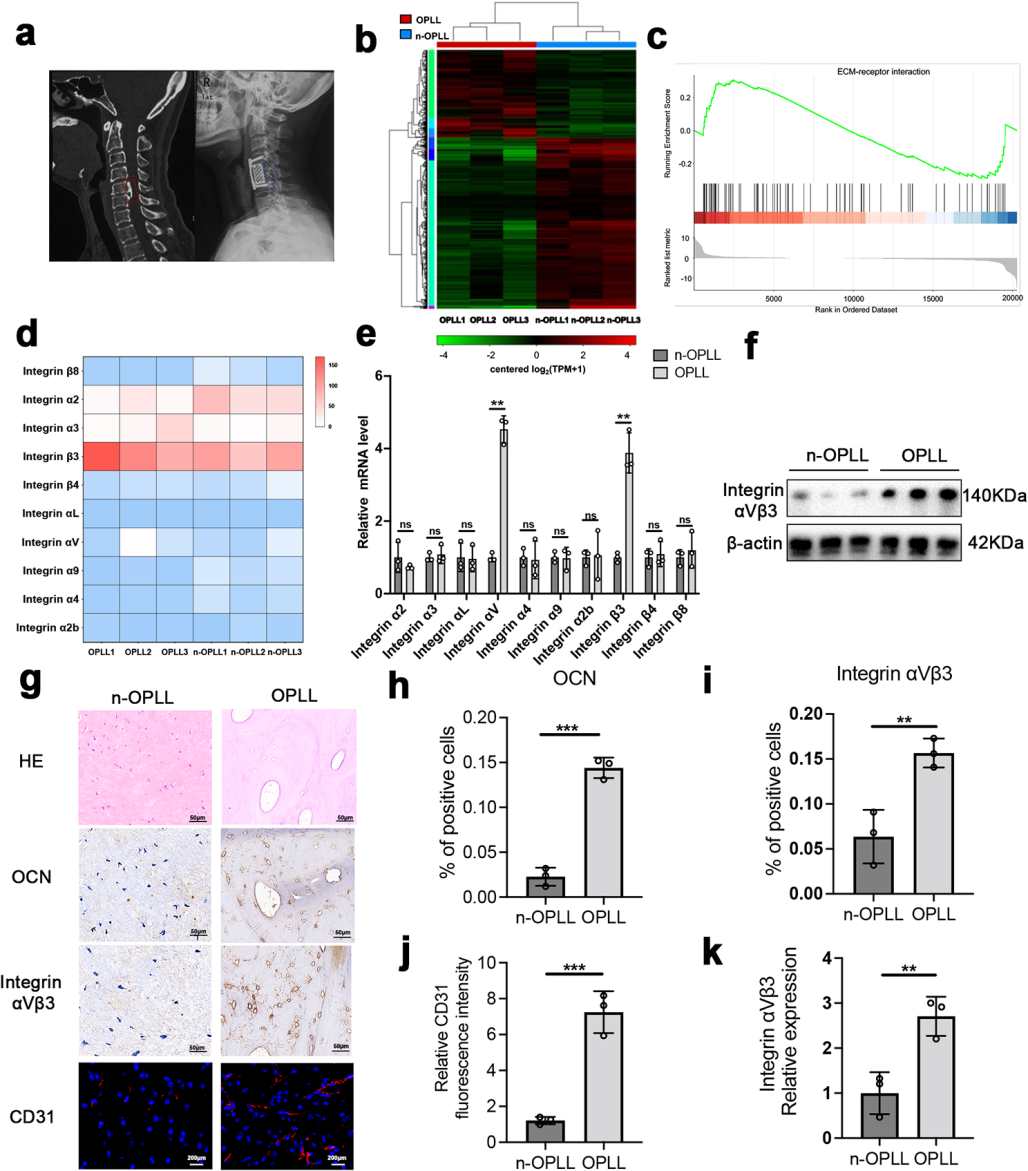
Integrin αVβ3 is upregulated in OPLL. **a** Typical preoperative CT and image of a patient with cervical OPLL (the red square at the left indicates OPLL of the cervical spine) and postoperative X-ray image indicating anterior en bloc resection of the cervical ossified posterior longitudinal ligament (the blue square at the right). **b** RNA-seq visualized by heatmap analysis. **c** GSEA plots illustrating upregulation of ECM receptor signaling pathway in LFs derived from patients with OPLL and non-OPLL. **d** Integrin subfamily molecule expression levels using the RNA-seq sequencing results visualized by heatmap analysis. **e** Relative mRNA expression levels of Integrin subfamily molecule. **f** The expression of integrin αVβ3 was detected by western blot. **g** HE staining, immunohistochemical analysis of OCN and integrin αVβ3, and immunofluorescence analysis of CD31 in ligament tissues from patients with OPLL and non-OPLL patients. **h** Quantitative analysis of OCN-positive cells in g. *n* = 3 per group. **i** Quantitative analysis of integrin αVβ3-positive cells in g. *n* = 3 per group. **j** Quantitative analysis of CD31-positive cells in g, *n* = 3 per group. **k** Quantitative analysis of integrin αVβ3 by western blot. *n* = 3 per group. All experimental data are presented as the mean value ± SD. Student’s t-test used in (**h**), (**i**), (**j**), and (**k**). *p < 0.05, **p < 0.01, ***p < 0.001

Pathological ligament tissues were collected from patients with OPLL during surgery. Typical preoperative and postoperative imaging features of the patients with OPLL and ligament samples obtained during surgery are shown in Fig. 1a and Figure S2. In addition, the normal ligaments of non-OPLL patients with spinal trauma were collected as a control group. As mentioned in the above method, LFs derived from patients with OPLL and non-OPLL were cultured separately for subsequent experiments. We postulated that differential gene expression in OPLL LFs contributes to ectopic ossification. To investigate this, we conducted RNA-seq analysis of OPLL LFs and non-OPLL LFs. The RNA-seq analysis unveiled 643 genes displaying differential expression in OPLL cells, with 218 genes upregulated and 425 genes downregulated (Fig. 1b). Following this, we performed Gene Set Enrichment Analysis (GSEA) on the sequencing data, which revealed that the ECM-receptor interaction pathway plays a crucial role in mediating the functional effects of differentially expressed genes (Fig. 1c). Many studies have shown that the integrin family plays an important role in ECM-receptor interaction, especially the activation of the integrin family proteins promoting osteogenic differentiation of mesenchymal stem cells (Marie 2013; Choi et al. 2020; Tang et al. 2021a). To elucidate the underlying molecular mechanism driving pathological ectopic bone formation in OPLL, we conducted a comprehensive analysis of integrin subfamily molecule expression levels using the RNA-seq sequencing results. The results revealed a significant upregulation of integrin subunits αV and β3 in OPLL LFs (Fig. 1d). Subsequently, total RNA was extracted from OPLL or n-OPLL LFs to validate the sequencing results. qPCR analysis demonstrated that αVβ3 exhibited the most significant upregulation in expression levels (Fig. 1e). Furthermore, western blotting showed higher expression of integrin αVβ3 in LFs derived from the patients with OPLL, further proving the positive role of integrin αVβ3 in OPLL progression (Fig. 1f, k). Osteocalcin (OCN) is secreted by osteoblasts and is considered a common marker of ossification. Immunohistochemical staining showed a significant number of OCN-positive cells in the OPLL samples, indicating that ossification of the posterior longitudinal ligament was occurring (Fig. 1g, second row). Consistent with our previous findings, immunohistochemical staining confirmed that integrin αVβ3 was upregulated during the ossification of ligament tissue in the patients with OPLL. Finally, we focused on the relationship between OPLL and angiogenesis, and immunofluorescence staining of CD31^+^ in ligament tissues confirmed that ECs had a positive role in ossification progression (Fig. 1g-j).

### LFs derived from OPLL patients have the potential for multidirectional differentiation

**Fig. 2:**
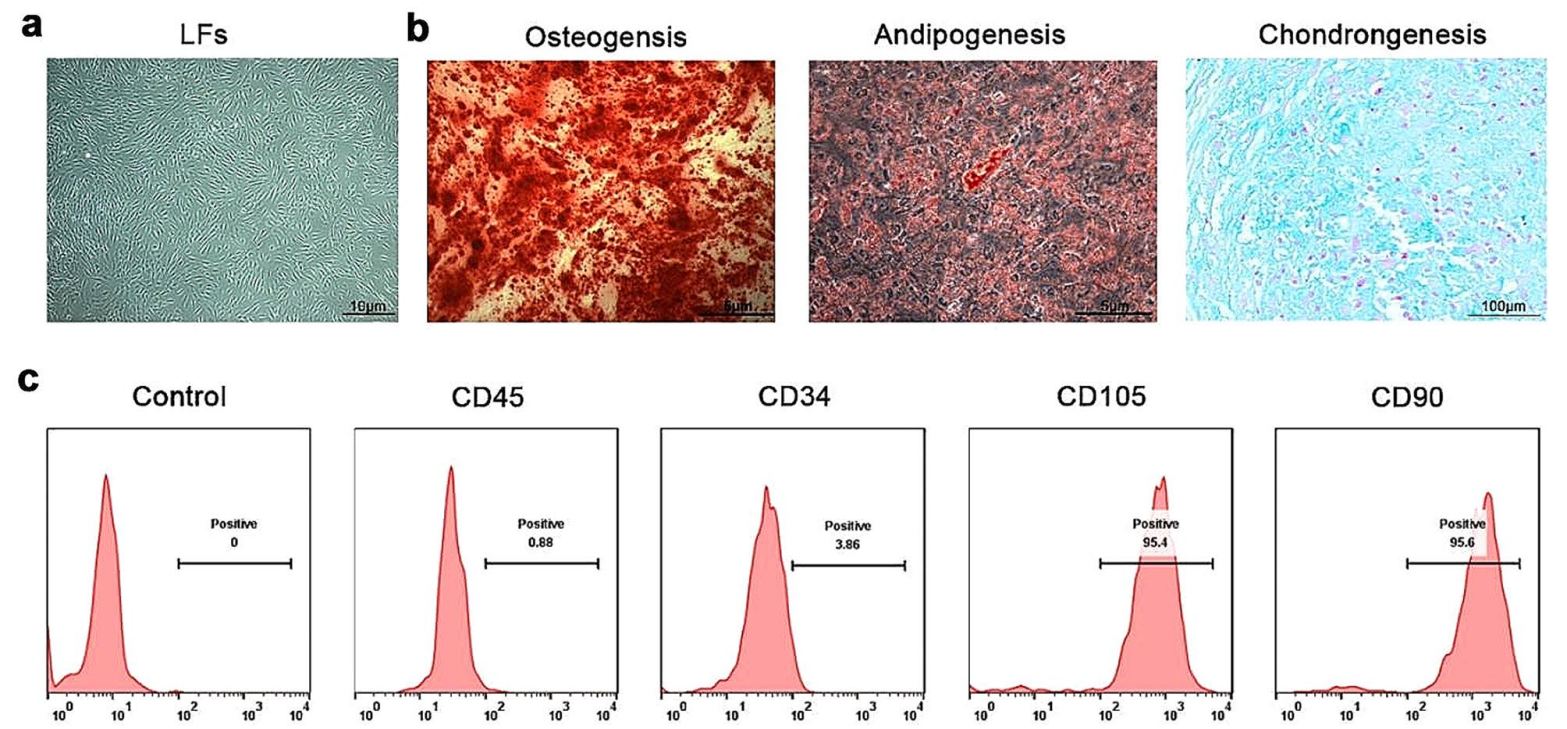
OPLL patient-derived LFs have the potential for multidirectional differentiation. **a** Cell morphology of LFs observed with a light microscope. **b** Staining with ARS, Oil Red O, and Alcian Blue was performed to test the osteogenic, adipogenic, and chondrogenic differentiation of LFs, respectively, and representative results are shown. **c** Flow cytometry to identify LF surface markers (negative markers: CD34, and CD45; positive markers: CD90 and CD105)

After 10 d of in vitro culture of ligament tissues from the patients with OPLL, well-grown LFs with spindle or flattened shapes emerged around the tissue pieces. The morphology of primary LFs is shown in Fig. 2a. To identify the multidirectional differentiation potential of the LFs, we induced primary LFs to undergo osteogenic, adipogenic, and chondrogenic differentiation. After induction, the cells were stained to verify the differentiation results, proving that primary LFs had trilineage differentiation ability (Fig. 2b). In addition, we detected the surface antigens, identifying high positive rates of CD90 and CD105 and low positive rates of CD34 and CD45 (Fig. 2c). Thus, the cultured primary cells were identified as LFs and possessed mesenchymal stem cell properties.

### C(RGDyk) peptide effectively inhibits the functions of integrin αVβ3 in LFs and ECs

**Fig. 3:**
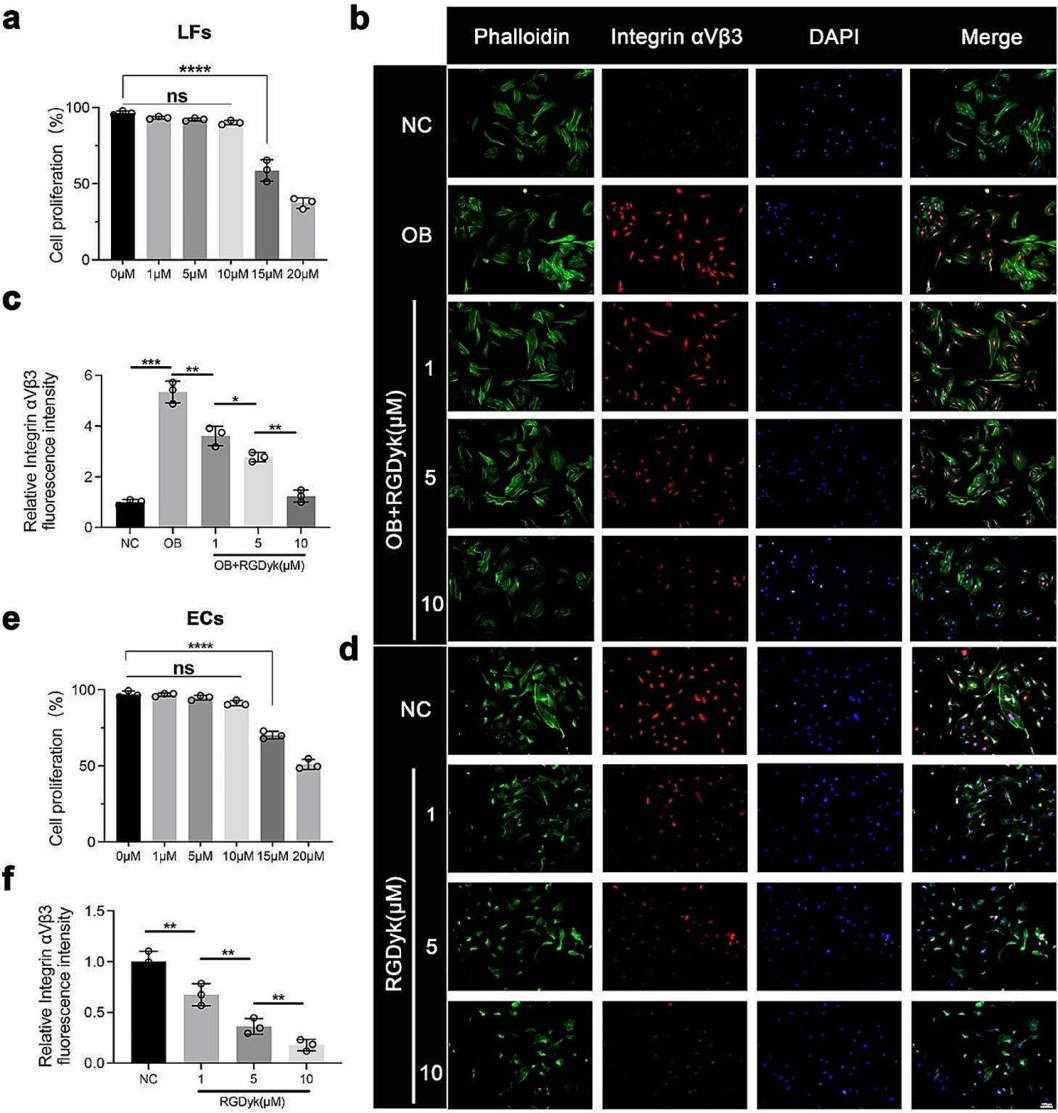
The c(RGDyk) peptide effectively inhibits the expression of integrin αVβ3 in LFs and ECs. **a** Cell proliferation of LFs when cultured with different concentrations of C(RGDyk) peptide. **b** Immunofluorescence of phalloidin and integrin αVβ3 in LFs. Scale bar:100 μm. **c** Quantitative analysis of integrin αVβ3 fluorescence intensity in LFs. **d** Cell proliferation of ECs when cultured with different concentrations of c(RGDyk) peptide. **e** Immunofluorescence of phalloidin and integrin αVβ3 in ECs. Scale bar:100 μm. **f** Quantitative analysis of integrin αVβ3 fluorescence intensity in ECs. All experiments were repeated three times. All experimental data are presented as the mean value ± SD. One-way ANOVA for multiple comparisons is used in (**a**), (**c**), (**e**), and (**f**). *p < 0.05, **p < 0.01, ***p < 0.001

To confirm the critical role of integrin αVβ3 in ectopic bone formation in OPLL, we administered an integrin αVβ3 antagonist-c(RGDyk) peptide to cultured LFs before and after osteogenic differentiation. Cell proliferation assays confirmed that concentrations higher than 10 µM affected LF proliferation (Fig. 3a). Immunofluorescence confirmed that the expression of integrin αVβ3 was upregulated after osteogenic induction culture of LFs. However, when LFs differentiated into osteoblasts (OB), the expression of integrin αVβ3 was significantly downregulated in the c(RGDyk) group (Fig. 3b, c). Next, we explored the effect of integrin αVβ3 on the biological activity of ECs in angiogenesis. Our results showed integrin αVβ3 was highly expressed in well-grown ECs. Similarly, cell proliferation assays and immunofluorescence confirmed that in ECs, 10 µM c(RGDyk) had a negligible effect on cell proliferation and effectively inhibited the expression of integrin αVβ3 (Fig. 3d-f).

### c(RGDyk) peptide suppresses the osteogenic differentiation of LFs

**Fig. 4:**
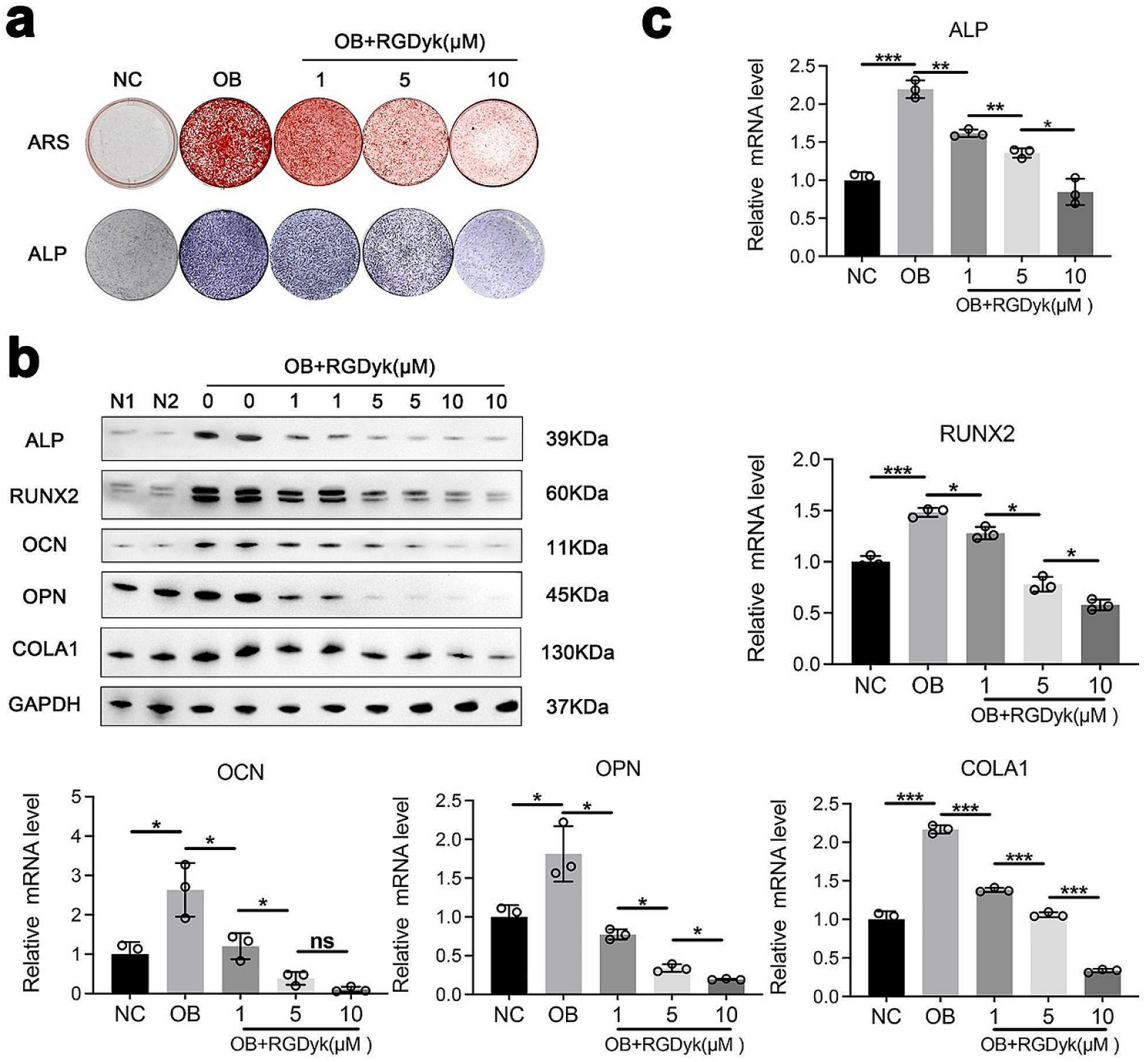
The c(RGDyk) peptide suppresses the osteogenic differentiation of LFs. **a** The inhibitory effect of the C(RGDyk) peptide on osteogenesis was confirmed by ARS and ALP staining. **b** Western blot analysis for the expression of osteogenesis-related genes. **c** The quantification of the expression of osteogenesis-related genes was detected by qPCR (*n* = 3, the latter group compared with the former group). GAPDH or β-actin was selected as the internal control. All experiments were repeated three times. All experimental data are presented as the mean value ± SD. One-way ANOVA for multiple comparisons is used in (c). *p < 0.05, **p < 0.01, ***p < 0.001

To further confirm the inhibitory effect of the c(RGDyk) peptide in OPLL, we studied the effect of the c(RGDyk) peptide on the osteogenic differentiation of LFs in vitro. We treated LFs with different concentrations of c(RGDyk) peptide, including 1 µM, 5 µM, and 10 µM. Briefly, c(RGDyk) peptide was added to the coculture with cells every two days when the osteogenic induction medium was replaced. Staining results showed that stimulation of LFs in osteogenic culture with c(RGDyk) resulted in attenuation of both ALP and ARS staining compared with that of an unstimulated osteogenic group (Fig. 4a). Similarly, q-PCR and western blot analysis results showed downregulation of the expression of osteogenesis-related genes in the c(RGDyk) inhibition group (Fig. 4b, c, and Additional file 1: Figure S3). Moreover, with increasing c(RGDyk) concentration, the inhibitory effect increased. In conclusion, we found that c(RGDyk) actively suppressed the osteogenic differentiation of LFs in vitro.

### c(RGDyk) peptide suppresses EC migration partly through the FAK/ERK signalling pathway

**Fig. 5:**
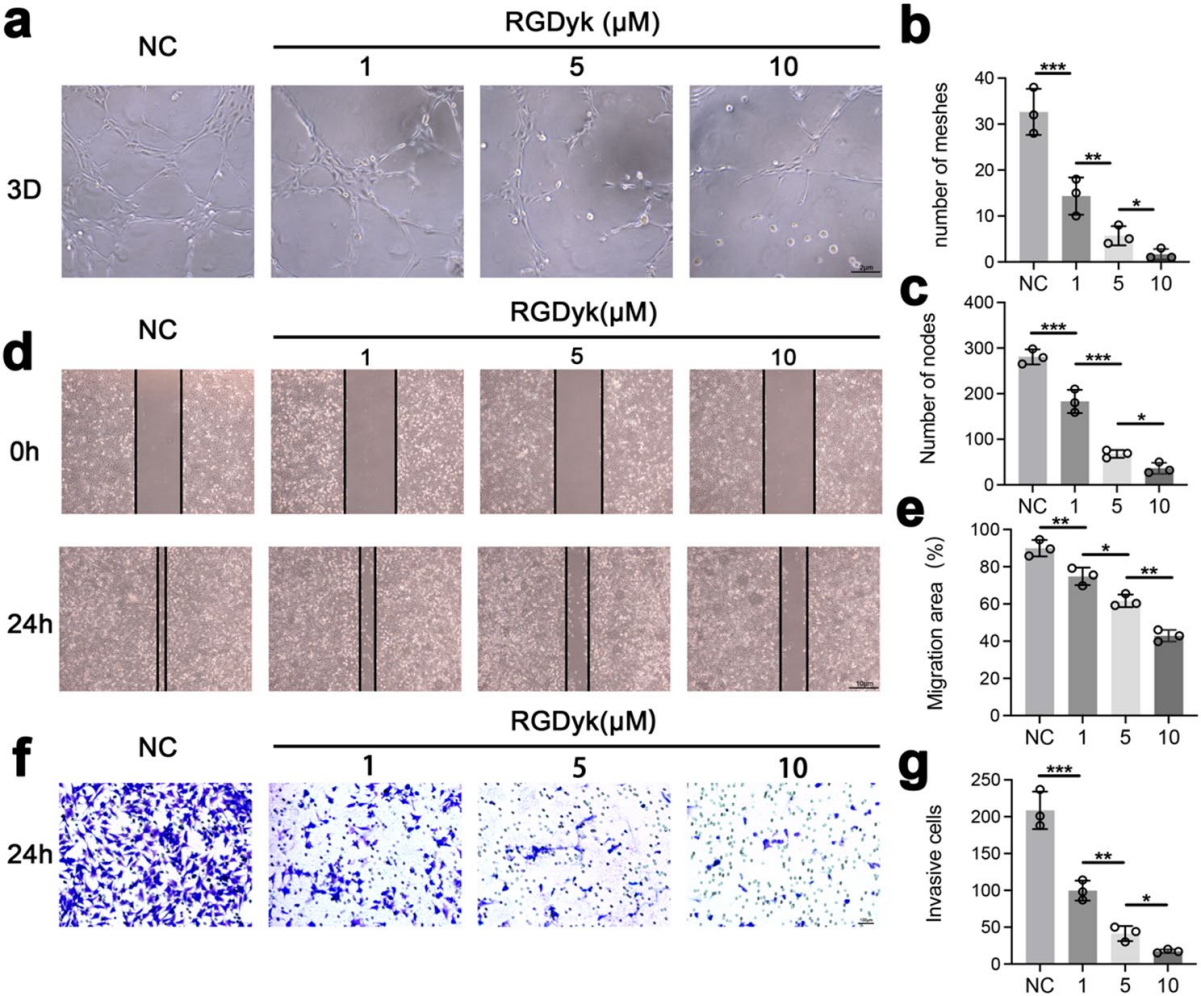
Investigation of the c(RGDyk) peptide on the angiogenesis-related phenotype of ECs in vitro. **a**-**c** Representative images and quantitative analysis of the tube formation assay in ECs. Scale bar:2 μm. **d**, **e** the scratch assay showing the motility of ECs treated with different concentrations of c(RGDyk) peptide. Scale bar:10 μm. **f** Representative images of EC migration assessed by Transwell assays. Scale bar:100 μm. **g** Quantitative analysis of the Transwell assay. All experiments were repeated three times. All experimental data are presented as the mean value ± SD. One-way ANOVA for multiple comparisons is used in (**b**), (**c**), (**e**), and (**g**). *p < 0.05, **p < 0.01, ***p < 0.001

Integrin αVβ3 is involved in vascular lumen formation and vascular network construction by regulating the adhesion and migration of EC in angiogenesis(Rocha et al. 2018). To determine whether c(RGDyk) inhibits angiogenesis in vitro, we performed a tube formation assay and confirmed that c(RGDyk) significantly reduced EC tube formation. The results showed that the ECs cultured on the pure 3D Matrigel produced a variety of different tubes, while the ECs cultured on Matrigel with c(RGDyk) rarely exhibited tube formation (Fig. 5a-c). Furthermore, to examine the role of c(RGDyk) in EC mobility, both transwell and scratch cell migration assays were performed. C(RGDyk) significantly inhibited more EC migration compared to that of the control group (Fig. 5d-g).

**Fig. 6:**
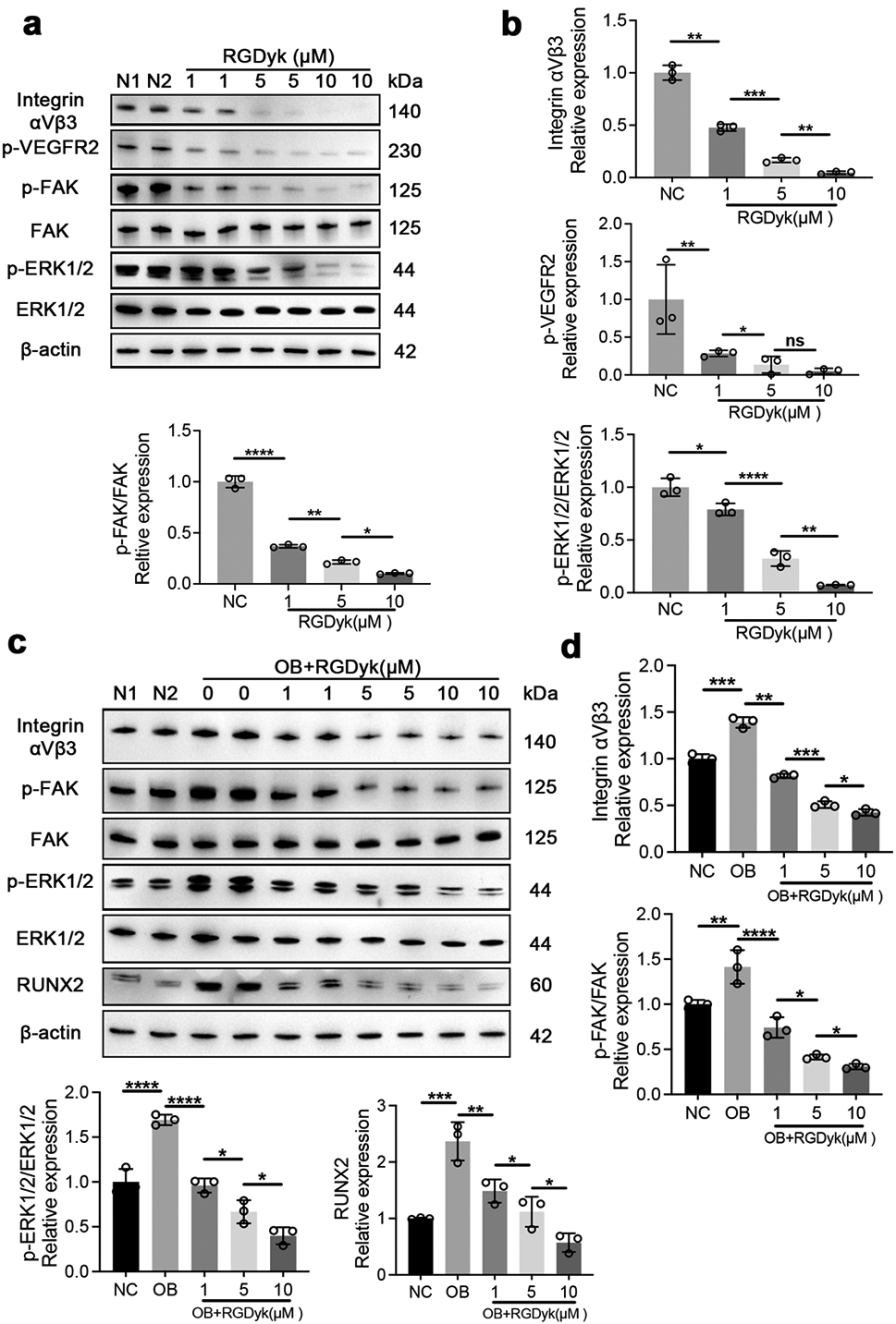
The c(RGDyk) peptide inhibits osteogenesis and angiogenesis by targeting integrin αVβ3 and regulating the FAK/ERK1/2 pathway. **a** and **b** Western blotting and quantitative analysis of the protein expression of the FAK/ERK1/2 pathway in ECs, including integrin αVβ3, p-VEGFR2, p-FAK, FAK, p-ERK1/2, and ERK1/2. **c** and **d** Western blotting and quantitative analysis of the protein expression of the FAK/ERK1/2 pathway in LFs, including integrin αVβ3, p-FAK, FAK, p-ERK1/2, ERK1/2, and RUNX2. β-actin was selected as the internal control. All experiments were repeated three times. All experimental data are presented as the mean value ± SD. One-way ANOVA for multiple comparisons is used in (**b**) and (**d**). *p < 0.05, **p < 0.01, ***p < 0.001

A close relationship exists between integrin αVβ3 and VEGFR2, both of which play an important role in angiogenesis (Somanath et al. 2009). However, EC exposure to the c(RGDyk) blocked the interaction of VRGFR2 with integrin αVβ3 (Fig. 6a). Angiogenesis and cell migration are known to be mediated by FAK activation (Zhao and Guan 2011). Western blotting analysis showed that c(RGDyk) significantly reduced the phosphorylation of FAK and ERK1/2 (Fig. 6a, b). Together, these results indicate that c(RGDyk) suppresses the FAK/ERK1/2-dependent migration of ECs by blocking the close relationship between integrin αVβ3 and VEGFR2 signalling.

### C(RGDyk) peptide inhibits osteogenesis by the integrin αVβ3/FAK/ERK/Runx2 axis

Both immunofluorescence and western blotting confirmed that integrin αVβ3 expression in LFs is enhanced under osteogenesis-inducing conditions. Integrin αVβ3 activation has been shown to affect osteogenic differentiation by activating FAK downstream (Oudart et al. 2017). Furthermore, FAK and ERK1/2 can induce bone phenotypes in mesenchymal stem cells (Mruthyunjaya et al. 2010). To better understand how c(RGDyk) inhibits the osteogenesis of LFs, we analyzed the related signalling pathway. Western blotting confirmed that a key transcription factor for osteogenic differentiation is RUNX2, which is activated downstream by integrin αVβ3 and FAK and ERK1/2 phosphorylation. In addition, c(RGDyk) inhibition of integrin αVβ3 decreased the expression of RUNX2 (Fig. 6c, d). Accordingly, c(RGDyk) directly targeted integrin αVβ3 and thus repressed the downstream FAK/ERK1/2/Runx2 signalling pathway. To further validate the downstream genes and signaling pathways regulated by Integrin αVβ3 in LF osteogenic differentiation, we constructed siRNAs targeting Integrin αV and Integrin β3, which were transfected into LF cells. Western blotting confirmed the successful transfection. Consistent with previous studies, Western blotting results demonstrated that compared to the control group, siRNA Integrin αV and siRNA Integrin β3 significantly inhibited the phosphorylation of FAK and ERK1/2, consequently downregulating the expression of RUNX2 (Additional file 1: Figure S4a, b). Therefore, integrin αVβ3 serves as a crucial upstream molecule promoting LF osteogenic differentiation, and inhibiting the expression of integrin αVβ3 can block the FAK/ERK1/2/Runx2 signaling pathway.

### c(RGDyk) peptide inhibits heterotopic bone formation in vivo

**Fig. 7:**
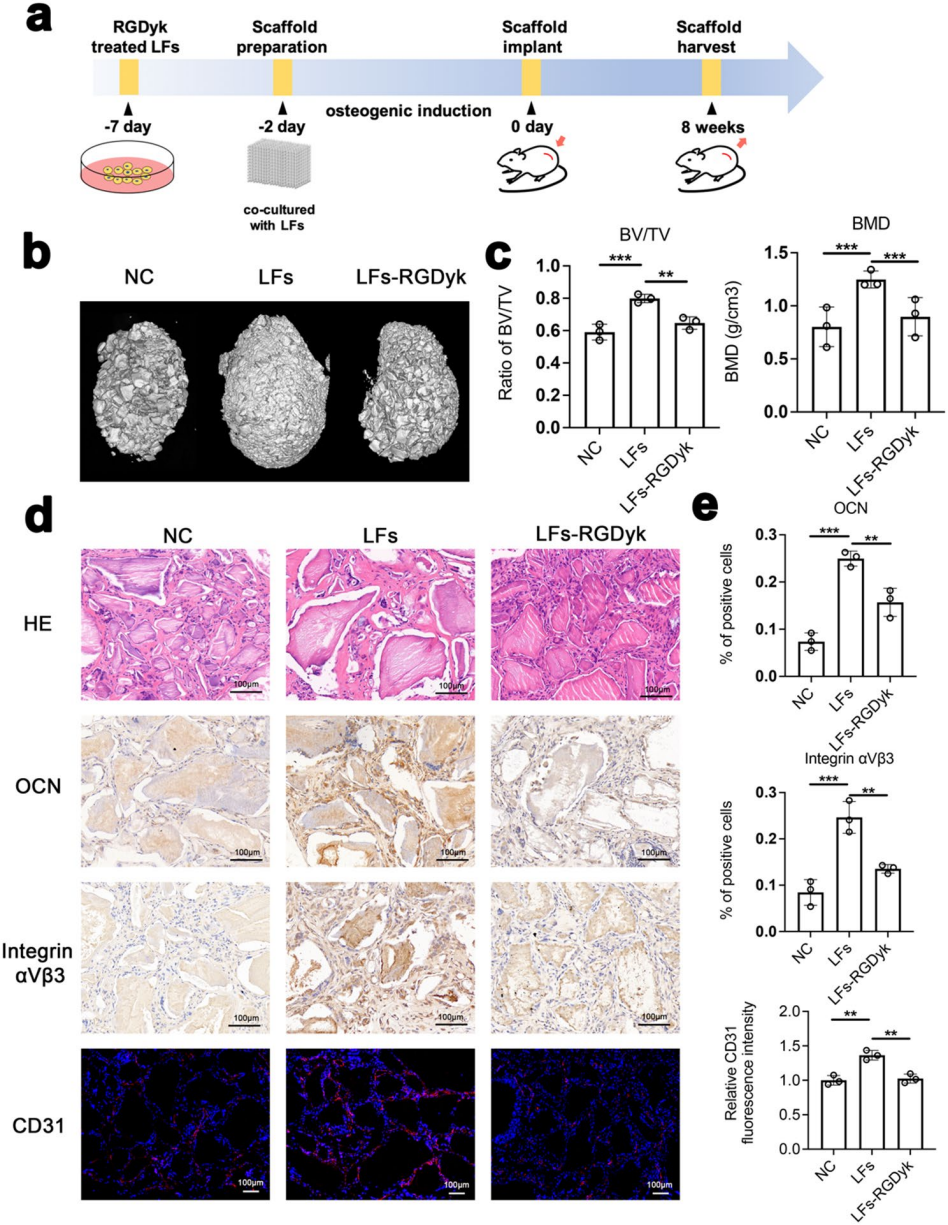
c(RGDyk) peptide inhibits heterotopic bone formation in vivo. **a** The procedures of the heterotopic bone formation assay in vivo. LFs were treated with c(RGDyk) peptide before implantation. **b** Representative 3D-reconstructed micro-CT images of a scaffold implanted in coculture with LFs after 8 weeks. **c** Micro-CT scan analysis of the BV/TV and BMD of scaffolds among different groups (*n* = 3). **d** HE staining, immunohistochemical analysis of OCN and integrin αVβ3, and immunofluorescence analysis of CD31 among different implanted scaffolds. **e** Quantitative analysis of OCN-positive, integrin αVβ3-positive, and CD31-positive cells in d. *n* = 3 per group. All experimental data are presented as the mean value ± SD. One-way ANOVA for multiple comparisons is used in (**c**) and (**e**). *p < 0.05, **p < 0.01, ***p < 0.001

To further analysis of the function of c(RGDyk), a heterotopic bone formation assay was performed subcutaneously in nude mice (Fig. 7a). LFs were treated with c(RGDyk) and cocultured with Bio-Oss Collagen for 48 h in osteogenic induction medium. The mixture of scaffold and LFs was then implanted into the subcutaneous space on the backs of nude mice and allowed to grow for 8 weeks. After the animals were sacrificed, the scaffolds were harvested, micro-CT was used to determine the bone mass and volume of the scaffolds, and the ectopic bone morphology was shown in the 3D reconstruction image. Compared to the LFs group, the LFs-c(RGDyk) group exhibited less bone mass formation (Fig. 7b). The results of micro-CT analysis, including the ratio of bone volume/tissue volume (BV/TV) and bone mineral density (BMD), were significantly increased in the LFs group, and the LFs-RGDyk group showed the opposite effect (Fig. 7c). Subsequently, we performed the histological examination and immunohistochemical detection of ectopic bone, and the analysis showed that the LFs group formed more ectopic bone and had more OCN-positive cells in the bone mass, while the LFs-RGDyk group formed less ectopic bone and showed fewer OCN-positive cells (Fig. 7d, e). Furthermore, the expression of integrin αVβ3 was analyzed by immunohistochemistry, and the results showed that in the ectopic bone treated with c(RGDyk), the rate of integrin αVβ3 positivity was significantly lower than that in the LFs group (Fig. 7d, e). Together, these results indicated that c(RGDyk) inhibited ossification in vivo, the effect of which was largely due to effectively inhibiting the expression of integrin αVβ3. Finally, the immunofluorescence assay showed that the number of blood vessels formed in the LFs-RGDyk group was significantly lower than that in the LFs group (Fig. 7d, e).

**Fig. 8:**
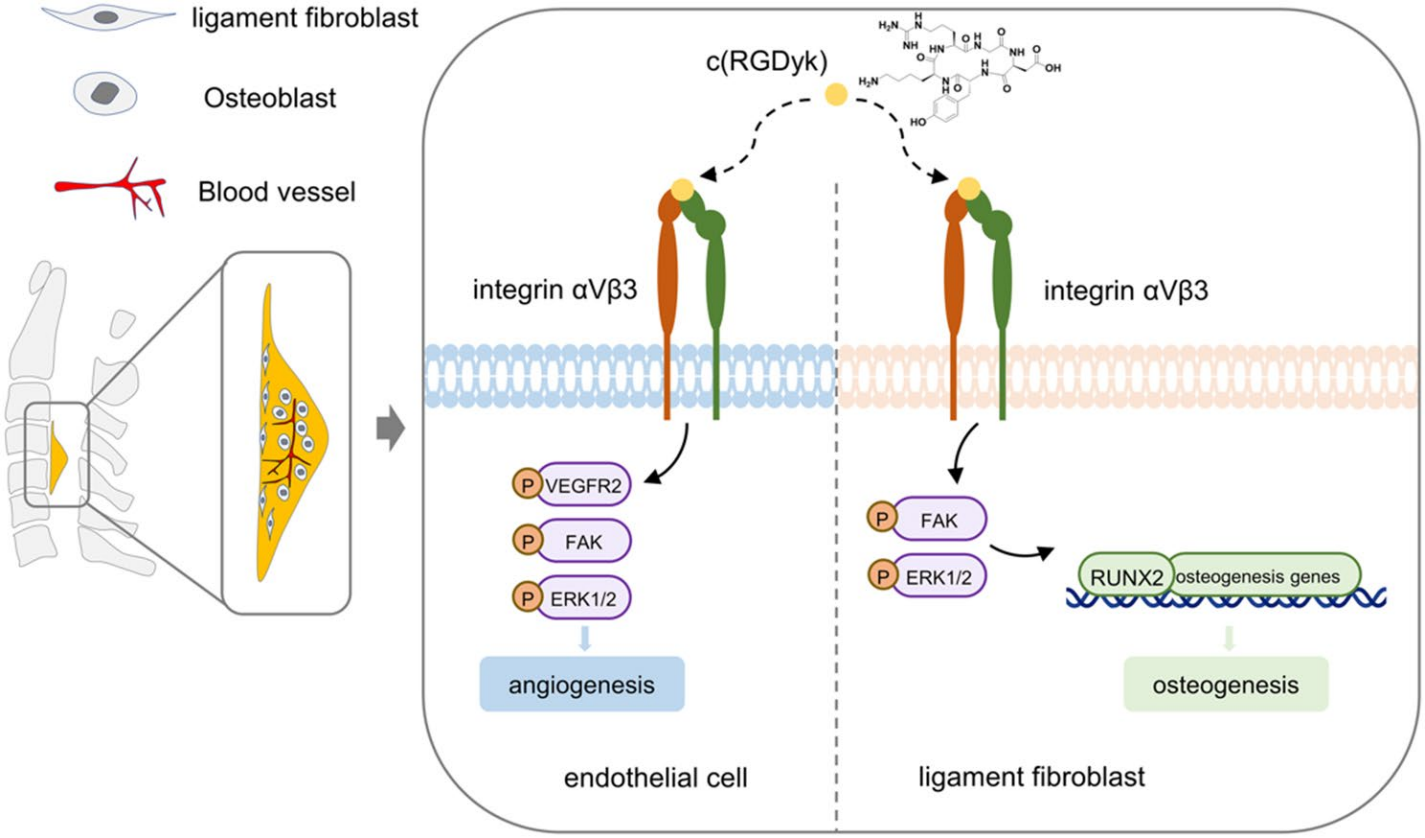
Schematic diagram of the mechanism by which c(RGDyk) suppressed integrin αVβ3 to inhibit OPLL

In summary, our results showed that c(RGDyk) bound to integrin αVβ3 on the surface of LFs and ECs, suppressed osteogenic differentiation of LFs and angiogenesis of ECs through the FAK/ERK pathway, and finally inhibited OPLL (Fig. 8).

## Discussion

Ectopic bone formation at the posterior longitudinal ligament is one of the prominent characteristics of OPLL and causes spinal cord compression, and motor and sensory dysfunction (Saetia, Cho 2011). Unfortunately, there is sometimes unsatisfactory treatment for OPLL. Because patients with early-stage OPLL usually do not exhibit neurological symptoms, many patients with continued symptoms have a high surgical risk due to the presence of large ossification of ligaments that occupy most of the space in the spinal canal and can cause serious complications, such as cerebrospinal fluid leakage or root nerve palsy (Boody et al. 2019). Although researches have been exploring the pathogenesis of OPLL to further develop nonsurgical treatment strategies, there are still no effective methods to inhibit or delay the progression of the disease. The process of OPLL is a complicated process involving various critical cell types, including osteoblasts, ligament fibroblasts, endothelial cells, ect. Numerous previous studies have shown ligament fibroblasts are responsible for the ossification process (Chen, Bi 2018, Su, Wei 2018, Cho, Kim 2017). Our results demonstrated that OPLL patient-derived ligament fibroblasts have mesenchymal stem cell properties and can differentiate into osteoblasts. In addition, we identified a transmembrane receptor protein, integrin αVβ3, that was abnormally upregulated in the ligaments of patients with OPLL. Furthermore, we found that integrin αVβ3 antagonist-c(RGDyk) dramatically suppressed the osteogenic differentiation of ligament fibroblasts, indicating the essential role of integrin αVβ3 in this pathological progression and the possibility of c(RGDyk) as a new nonsurgical treatment strategy.

The different biomechanics of the spine affect the pathogenesis of ligament ossification, and mechanical stress stimulation plays an important role in the occurrence and development of ligament ossification (Won et al. 2022; Chen et al. 2017; Tanno et al. 2003). Chen et al. (Zhang et al. 2014)showed that ligament cells derived from patients with OPLL are more sensitive to mechanical stress than non-OPLL patient-derived ligament cells, and ligament cells derived from patients with OPLL have stronger osteogenic differentiation under stress stimulation. In addition, according to the characteristics of clinical cases, the incidence of OPLL is related to the stress concentration of the cervical spine. However, the exact mechanism of OPLL pathogenesis caused by an abnormal stress microenvironment is rarely studied. The extracellular matrix (ECM) determines the various properties of tissue microenvironments, such as stress, resilience, and adhesion (Kechagia, Ivaska 2019, Romani et al. 2021; Humphrey et al. 2014). Interestingly, Interestingly, the mechanical stress properties of tissues strongly affect the adipogenic, osteogenic, and chondrogenic differentiation of mesenchymal stem cells (Yu et al. 2021; Sachs 2010; Sarraf et al. 2011).

Integrin αVβ3 is a cell adhesion and signalling protein involved in mediating cell-ECM interactions as a receptor that is essential for a wide range of biological functions (Zhang et al. 2021). Moreover, integrin αVβ3-mediated adhesion of osteoblasts to ECM plays a crucial role in regulating the functional activities of osteoblasts. Research has shown that activation of the integrin αVβ3-microfilament axis by cyclic tensile stress promotes osteogenesis of human fibroblasts (Peng, Qu 2021). Our results were consistent with these studies. As we have shown, in vitro studies have demonstrated that integrin αVβ3 expression is increased after osteogenic induction ligament cells derived from patients with OPLL. Focal adhesion kinase (FAK) is a highly conserved cytoplasmic tyrosine kinase, which is a key signalling molecule in the integrin-mediated extracellular-to-intracellular signalling pathway (Zhao and Guan 2011; Alanko and Ivaska 2016). Moreover, it has been shown that phosphorylated FAK can receive signals from integrin αVβ3 and activate a series of signalling pathways in cells to regulate cell growth and differentiation (Fu, Zhang 2020, Luo et al. 2018). We found the same phenomenon in LFs: a high-affinity state of integrin αVβ3 can activate FAK phosphorylation, thereby activating the ERK1/2 pathway to promote the expression of the key osteoblast gene RUNX2. Based on these results, we suggest that integrin αVβ3 plays a dominant role in the osteogenic differentiation of LFs.

The formation of new bone needs to be provided nutrients through blood vessels, and blood vessels of bone tissue provide a solid material basis for its homeostasis maintenance. Our results show that the ligament tissues of patients with OPLL had more CD31-positive neovascularization than the ligament tissues of non-OPLL patients. Thus, angiogenesis is an indispensable part of the formation of ectopic new bone formation at the posterior longitudinal ligament. Angiogenesis is driven by endothelial cell (EC) proliferation, migration, and tube formation, during which ECs collectively coordinate movement through remodelling interactions with the vascular microenvironment and contact between ECs (Eelen et al. 2018; Potente and Carmeliet 2017). Many studies have found that integrin αVβ3 is highly expressed in various malignant tumour tissues and that the expression level of integrin αVβ3 promotes the formation of new blood vessels, which is closely related to the biological behaviour of tumour invasion and distant organ metastasis (Atkinson et al. 2014; Robinson and Hodivala-Dilke 2011). As shown, integrin αVβ3 is clearly expressed in both LFs and ECs. In addition, the crosstalk relationship between integrin αVβ3 and VEGFR2 is important for the angiogenic activities of ECs, including migration and tube formation (Somanath, Malinin 2009). Our results also show that in ECs, integrin αVβ3 promotes FAK/ERK1/2-dependent EC migration by activating the VEGFR2 receptor to enhance the crosstalk between them.

In summary, integrin αVβ3 plays an important role in both osteogenesis and angiogenesis in the progression of OPLL. Therefore, a drug that can target the recognition of integrin αVβ3 and inhibit its abnormal expression is urgently needed to attenuate OPLL progression. Integrins serve to transmit signals from inside the cell to the outside, or from the outside to the inside, achieved through binding with intracellular cytoskeletal proteins or extracellular ligands (Koh et al. 2019). Integrin αVβ3 belongs to the RGD receptor subfamily and is one of the earliest and most extensively studied subtypes. The binding of integrins to ligands is mediated by recognizing the RGD sequence on the ligand. Therefore, the selective binding of peptides carrying the RGD sequence to integrin αVβ3 may achieve targeted inhibition of integrin αVβ3 (Santulli et al. 2011; Silva et al. 2020; Ariyani et al. 2022; Askew et al. 2018). Based on the characteristics of integrins mentioned above, there are currently anti-tumor drugs targeting integrins that have entered clinical trials (Gu et al. 2023; Kerr et al. 2002). Here, we applied the c(RGDyk) peptide, which exhibits a high affinity for integrin αVβ3 and inhibits the expression of integrin αVβ3 in both LFs and ECs. Cell proliferation assays confirmed that concentrations higher than 10 µM affected LF and ECs proliferation (Fig. 3a, e). In addition, we demonstrated the best inhibitory effect of the 10 µM c(RGDyk) peptide on osteogenesis and angiogenesis in vitro and in vivo. Interestingly, we also found that c(RGDyk) decreased the expression of integrin αVβ3. We hypothesize that c(RGDyk) may have a role in decreasing the protein stability of integrin αVβ3, which needs more investigation in the future. Moreover, western blots clarified specific molecular signalling pathways; for example, the c(RGDyk) peptide directly targeted integrin αVβ3, which inhibits osteogenesis via the FAK/ERK/RUNX2 axis and EC migration through the pVEGFR2/FAK/ERK axis. Therefore, we suggest that integrin αVβ3 plays an important role in OPLL. C(RGDyk), as an integrin αVβ3 antagonist, can effectively inhibit its downstream signal crosstalk.

However, our study has certain limitations. Although the in vitro cellular-level validation in this study is relatively comprehensive, the selection of the OPLL animal model and the validation at the in vivo animal level are limited. As most scholars consider OPLL to be a genetic disease, a large number of studies currently choose tip-toe walking (ttw) mice as the animal model of OPLL (Nakajima et al. 2020; Ichikawa et al. 2021). These mice, through Enpp1 knockout, exhibit spontaneous ossification of multiple joints and ligaments, including spontaneous ossification of the posterior longitudinal ligament, manifested as a characteristic tip-toe walking gait. Therefore, we plan to use ttw mice in subsequent studies to evaluate the targeting value of integrin αvβ3. When the ttw mice exhibit symptoms of spinal cord compression, we plan to intravenously inject the integrin αvβ3-targeting inhibitor c(RGDyk) peptide and observe the progression of ossification after inhibiting integrin αvβ3, followed by a series of subsequent result analyses. Therefore, our future research will provide experimental support and theoretical basis for the c(RGDyk) peptide as a novel treatment strategy.

## Conclusions

The present study confirms the key role of integrin αVβ3 in OPLL progression. Integrin αVβ3 antagonist-c(RGDyk) peptide can effectively inhibit osteogenesis and angiogenesis by targeting integrin αVβ3 and regulating the FAK/ERK pathway. In summary, integrin αVβ3 might be a potential therapeutic target for OPLL, and c(RGDyk) peptide as a targeted inhibitor might be used as a new therapeutic strategy for OPLL in the future.

### Electronic supplementary material

Below is the link to the electronic supplementary material.

Supplementary Material 1

## Data Availability

Data from the experiment are available on request from the corresponding author.

## Abbreviations

OPLL: Ossification of the Posterior Longitudinal Ligament
LFs: Ligaments Fibroblasts
ECs: Endothelial Cells
c(RDGyk) peptide: Cyclo (Arg-Gly-Asp-D-Tyr-Lys) Peptide
IF: Immunofluorescence
IHC: Immunohistochemical
ALP: Alkaline Phosphatase
RUNX2: Runt-related Transcription Factor 2
OCN: Osteocalcin
OPN: Osteopontin
COLA1: Collagen 1
BV/TV: Bone Volume/Tissue Volume
BMD: Bone Mineral Density
ECM: Extracellular Matrix
FAK: Focal Adhesion Kinase
OB: Osteoblasts
