# Supplementary material for: Integrin αVβ3 antagonist-c(RGDyk) peptide attenuates the progression of ossification of the posterior longitudinal ligament by inhibiting osteogenesis and angiogenesis
Source: Mol Med. 2024 May 2;30:57. doi: 10.1186/s10020-024-00822-x (PMC11067224; doi:10.1186/s10020-024-00822-x)
Supplement: Supplementary file 1 — Supplementary Material 1 [file 10020_2024_822_MOESM1_ESM.docx]

**Supplementary data**

# Integrin αVβ3 antagonist-c(RGDyk) peptide attenuates the progression of ossification of the posterior longitudinal ligament by inhibiting osteogenesis and angiogenesis

#

Xiangwu Geng**^1†^**, Yifan Tang**^1†^**, Changjiang Gu**^1†^**, Junkai Zeng^1^, Yin Zhao^1^, Quanwei, Zhou^1^, Lianshun Jia^1^, Shengyuan Zhou**^1^***, Xiongsheng Chen**^1^***

**^1^**Spine Center

Department of Orthopaedics

Changzheng Hospital

Naval Medical University (Second Military Medical University)

Shanghai 200003, P. R. China

***** Correspondence: zsy32@163.com; chenxiongsheng@vip.sohu.com

**^†^** *Xiangwu Geng, Yifan Tang, and Changjiang Gu contributed equally to this work.*

**Figure S1.** **Preoperative,** **intraoperative, and** **postoperative images of animal models.**

**
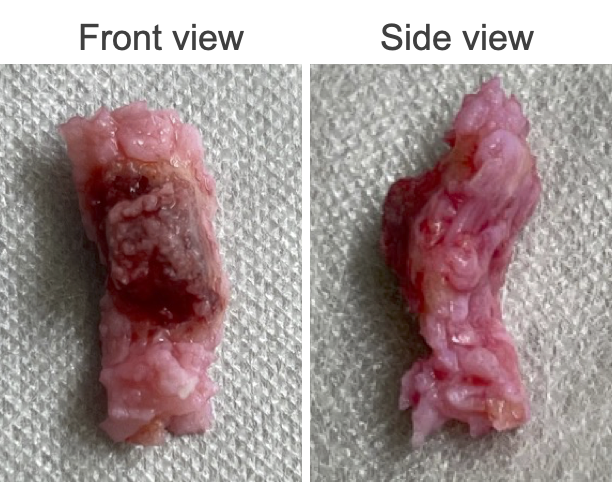
**

**Figure S2. Representative picture of OPLL sample.**


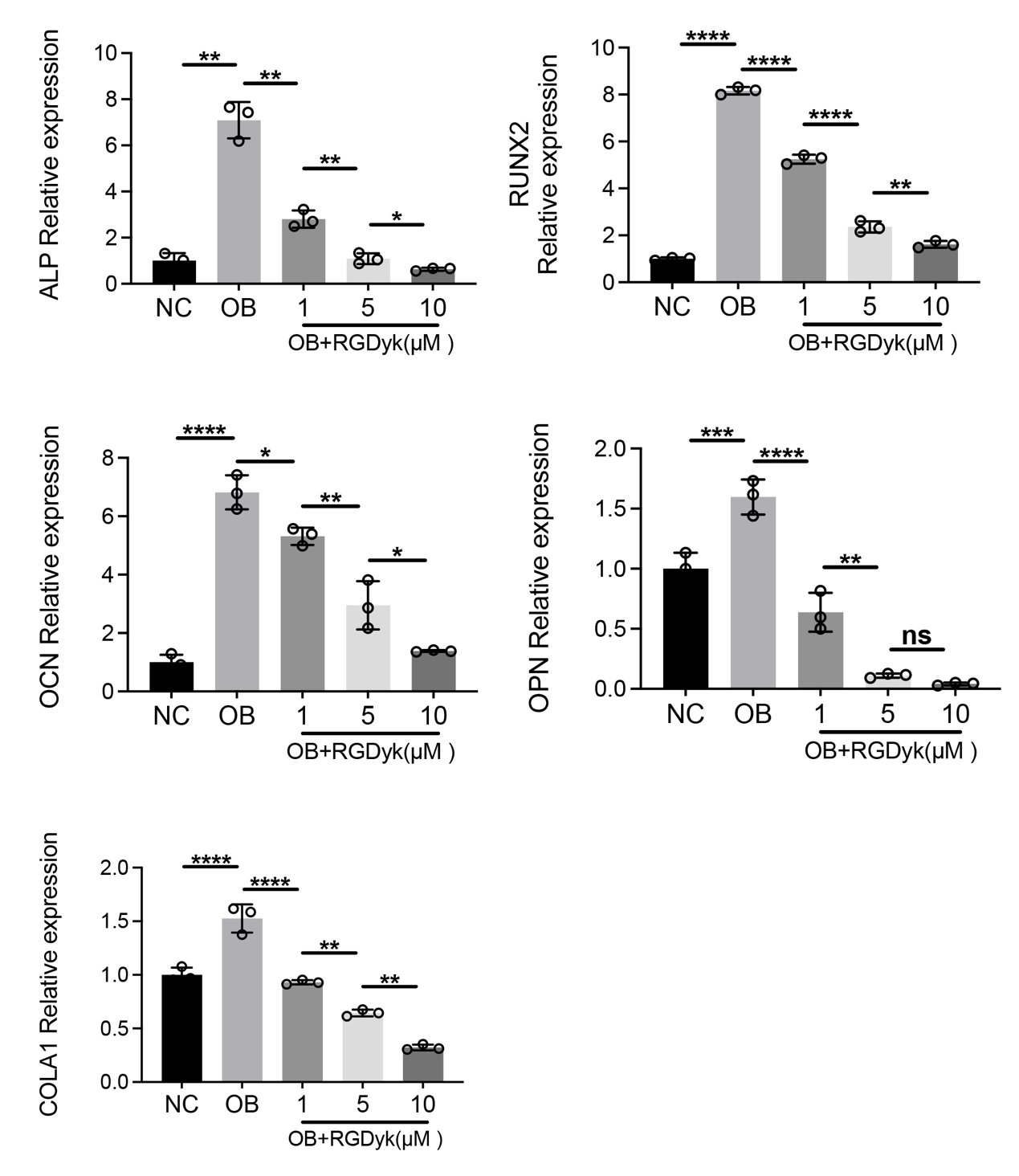


**Figure S3.** The quantification of the expression of osteogenesis-related genes was detected by western blots. All experimental data are presented as the mean value ± SD. One-way ANOVA for multiple comparisons is used in Figure S3. *p<0.05, **p<0.01, ***p<0.001.


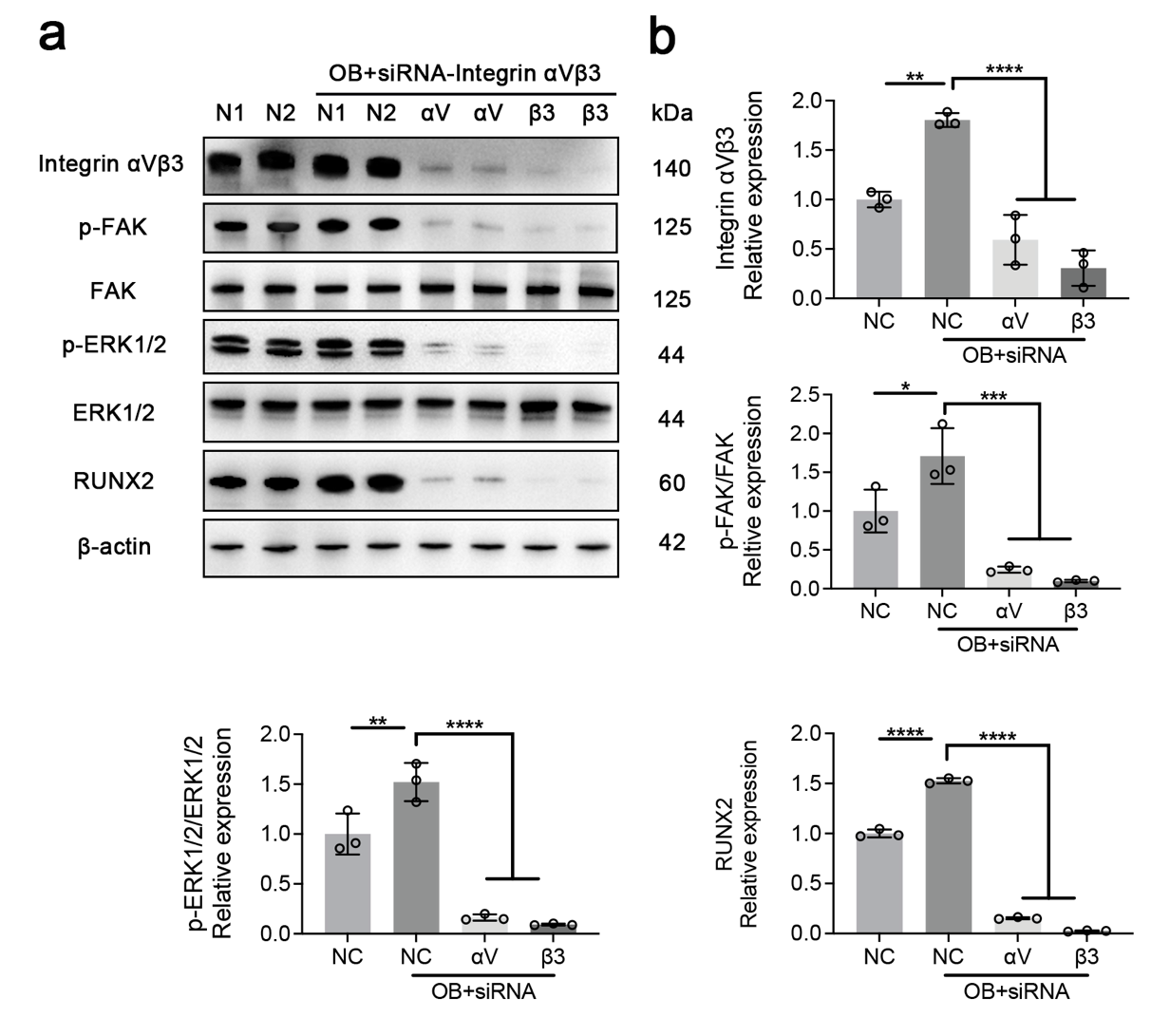


**Figure S4.** siRNA Integrin αVβ3 inhibits osteogenesis by regulating the FAK/ERK1/2/Runx2 pathway. **a** and **b** Western blotting and quantitative analysis of the protein expression of the FAK/ERK1/2 pathway in LFs, including integrin αVβ3, p-FAK, FAK, p-ERK1/2, ERK1/2, and RUNX2. β-actin was selected as the internal control. All experiments were repeated three times. All experimental data are presented as the mean value ± SD. One-way ANOVA for multiple comparisons is used in (b). *p<0.05, **p<0.01, ***p<0.001.


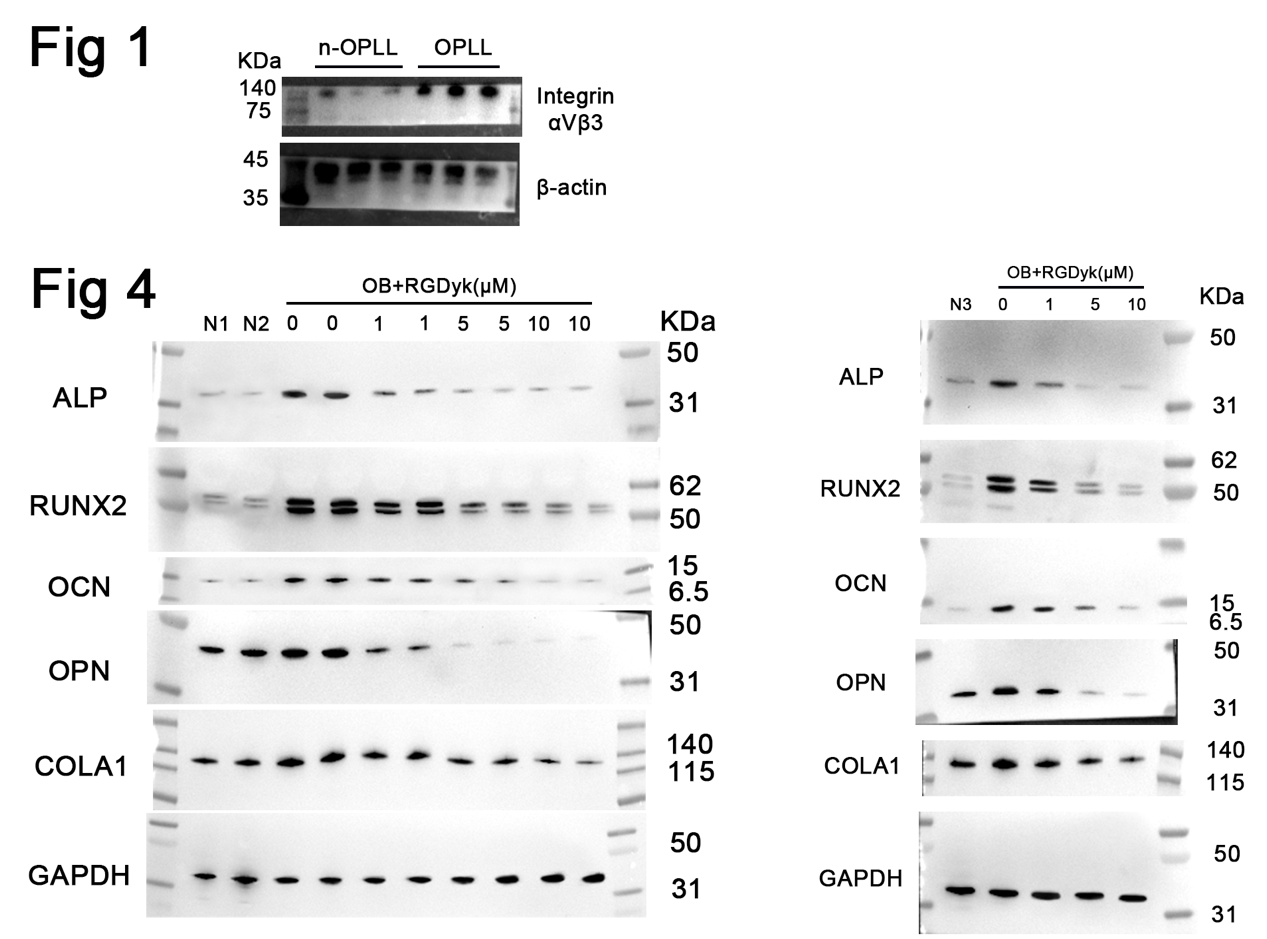


**Figure S5.** Original immunoblot pictures of Fig 1 and Fig 4.


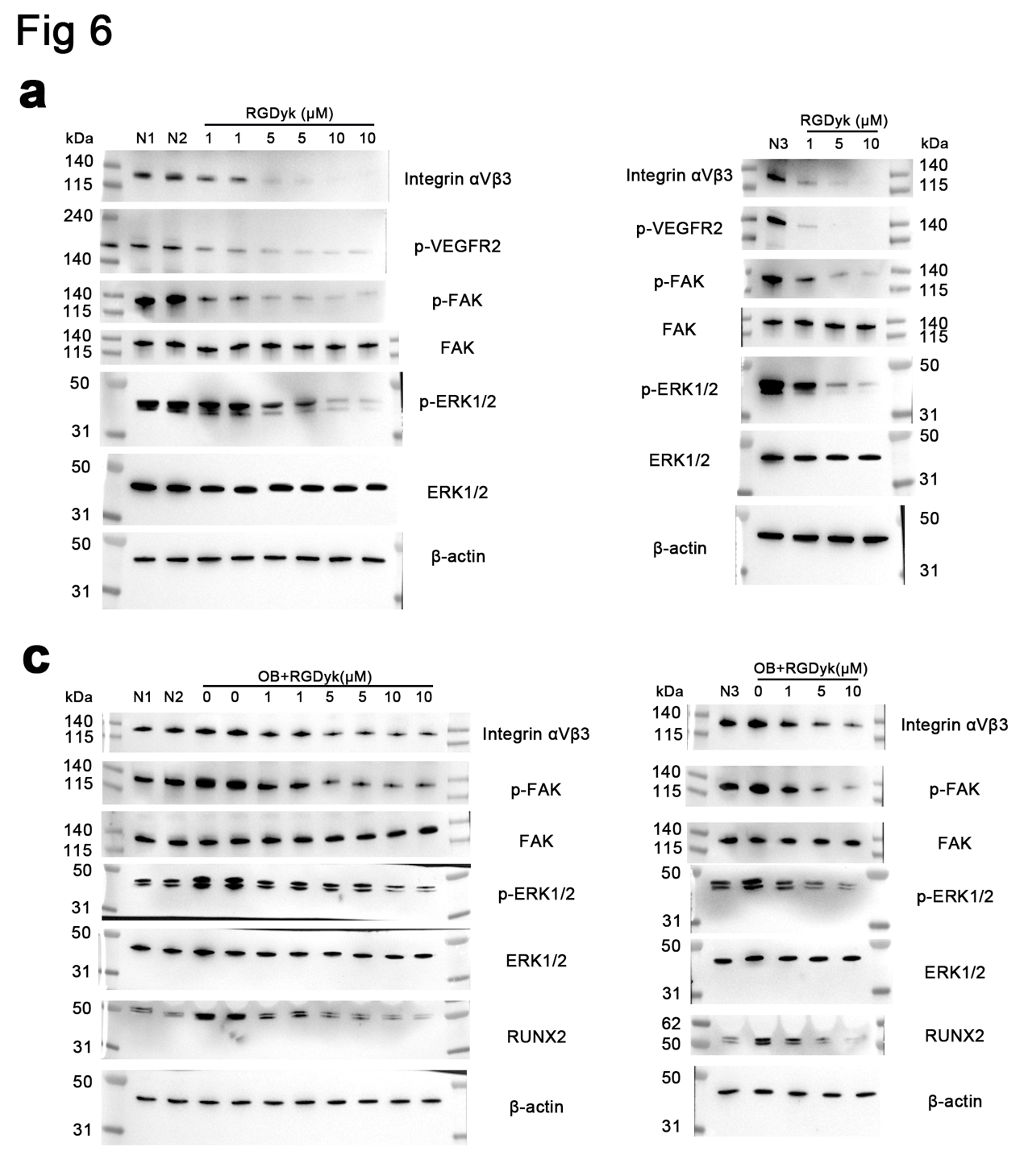


**Figure S6.** Original immunoblot pictures of Fig 6.


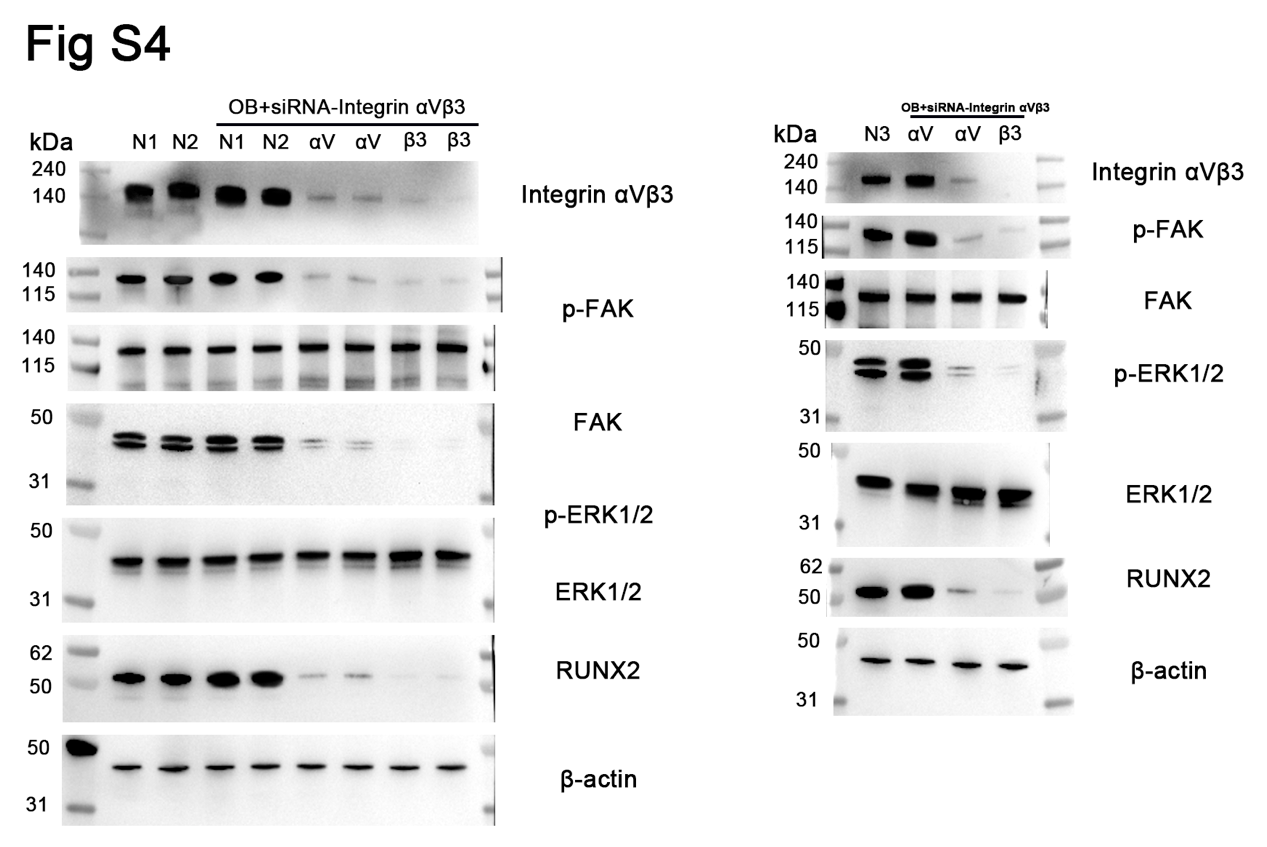


**Figure S7.** Original immunoblot pictures of Fig S4.

**Table S1. Characteristics of patient**

| **Group** | **Sex** | **Age** | **Diagnosis** | **Surgical level** |
| --- | --- | --- | --- | --- |
| OPLL 1 | male | 54 | OPLL | C3 |
| OPLL 2 | male | 65 | OPLL | C5 |
| OPLL 3 | male | 59 | OPLL | C4 |
| OPLL 4 | female | 60 | OPLL | C5 |
| OPLL 5 | female | 57 | OPLL | C4 |
| OPLL 6 | male | 67 | OPLL | C5 |
| OPLL 7 | male | 75 | OPLL | C4 |
| OPLL 8 | female | 70 | OPLL | C4 |
| non-OPLL 1 | male | 45 | cervical spine trauma | C4 |
| non-OPLL 2 | male | 50 | cervical spine trauma | C3 |
| non-OPLL 3 | male | 53 | cervical spine trauma | C4 |
| non-OPLL 4 | female | 55 | cervical spine trauma | C4 |
| non-OPLL 5 | female | 59 | cervical spine trauma | C4 |
| non-OPLL 6 | male | 60 | cervical spine trauma | C5 |
| non-OPLL 7 | male | 63 | cervical spine trauma | C4 |
| non-OPLL 8 | female | 65 | cervical spine trauma | C5 |
| ***P* value** | **0.654** | **0.716** | **-** | **0.576** |

OPLL: ossification of the posterior longitudinal ligament

**Table S2. The sequences of qPCR primers.**

| Primer Name |  | Sequence（5′-3′） | Length | Gene | Gene ID | product length | Tm(℃) |
| --- | --- | --- | --- | --- | --- | --- | --- |
| αv | F | GCTGTCGGAGATTTCAATGGT | 21 | ITGAV | 3685 | 136 bp | 58.64 |
|  | R | TCTGCTCGCCAGTAAAATTGT | 21 |  |  |  | 58.22 |
| α3 | F | CTACCACAACGAGATGTGCAA | 21 | ITGA3 | 3675 | 102 bp | 58.59 |
|  | R | CCGAAGTACACAGTGTTCTGG | 21 |  |  |  | 58.60 |
| α2 | F | CCTTGAAGCCTATTCTGAGACTGCC | 25 | ITGA2 | 3673 | 214 bp | 63.15 |
|  | R | AATTCCAGTGTTGTATGCACTTTCCC | 26 |  |  |  | 62.19 |
| α4 | F | AGCCCTAATGGAGAACCTTGT | 21 | ITGA4 | 3676 | 167 bp | 58.72 |
|  | R | CCAGTGGGGAGCTTATTTTCAT | 22 |  |  |  | 58.36 |
| α2b | F | GTTCAACGTGTCCTCCCTCC | 20 | ITGA2B | 3674 | 194bp | 60.32 |
|  | R | TGAAGAAGCCGACCTTCCAC | 20 |  |  |  | 59.97 |
| α9 | F | TCGTTCTTCGGCTACGCAG | 19 | ITGA9 | 3680 | 243 bp | 60.15 |
|  | R | CCCCATCCACTCATCATCGC | 20 |  |  |  | 60.60 |
| αL | F | GTCAGCTCATCATCCGAAACTG | 22 | FASTK | 10922 | 72 bp | 59.39 |
|  | R | AGACTGCAAGGTGCAGACACA | 21 |  |  |  | 62.19 |
| β3 | F | AATCTCCTGTGCATCACATTTCT | 23 | ITGB3BP | 23421 | 139 bp | 58.40 |
|  | R | TTCATAGCTGTCAAGATGACGTG | 23 |  |  |  | 58.82 |
| β4 | F | TGTCCATCCCCATCATCCCT | 20 | ITGB4 | 3691 | 106bp | 60.03 |
|  | R | CCCGATGGAGAGCGTAGAAC | 20 |  |  |  | 59.97 |
| β8 | F | TGGTGCTCTCAGGAGAGGTT | 20 | ITGB8 | 3696 | 187 bp | 55.00 |
|  | R | CTGGTCGTAGGTGGATGGTG | 20 |  |  |  | 60.00 |
| ALP | F | GTGGCAACTCTATCTTTGGTCTG | 23 | ALPL | 249 | 157 bp | 59.31 |
|  | R | GCCTGGTAGTTGTTGTGAGCA | 21 |  |  |  | 60.38 |
| RUNX2 | F | GCGCATTCCTCATCCCAGTA | 20 | RUNX2 | 860 | 176 bp | 59.89 |
|  | R | GGCTCAGGTAGGAGGGGTAA | 20 |  |  |  | 60.03 |
| OCN | F | AGGGCAGCGAGGTAGTGAAGAG | 22 | BGLAP | 1E+08 | 135 bp | 63.75 |
|  | R | GGTCAGCCAACTCGTCACAGTC | 22 |  |  |  | 63.34 |
| OPN | F | GTCAAAATCTAAGAAGTTTCGCAG | 25 | SPP1 | 6696 | 153 bp | 56.86 |
|  | R | CTGTCCCAATCAGAAGGCG | 19 |  |  |  | 58.23 |
| COLA1 | F | CGAAGACATCCCACCAATCA | 20 | COL1A1 | 1277 | 127 bp | 57.60 |
|  | R | GATCACGTCATCGCACAACA | 20 |  |  |  | 58.93 |
| β-actin | F | TAGTTGCGTTACACCCTTTCTTG | 23 | ACTB | 60 | 151 bp | 59.19 |
|  | R | TCACCTTCACCGTTCCAGTTT | 21 |  |  |  | 59.79 |

**Table S3. siRNA sequences.**

| siRNA name | Sequences (5'-3') |
| --- | --- |
| si-ITGαV-a | CTCTGTTGTATATCCTTCATT |
| si-ITGαV-b | GTGAGGTCGAAACAGGATAAA |
| si-ITGαV-c | CGACAGGCTCACATTCTACTT |
| si-ITGβ3-a | CCACGTCTACCTTCACCAATA |
| si-ITGβ3-b | GATGCAGTGAATTGTACCTAT |
| si-ITGβ3-c | CCTTAGCCTTTGTCCCAGAAT |
